# Understanding Human Decision Making in an Interactive Landslide Simulator Tool via Reinforcement Learning

**DOI:** 10.3389/fpsyg.2020.499422

**Published:** 2021-02-10

**Authors:** Pratik Chaturvedi, Varun Dutt

**Affiliations:** ^1^Applied Cognitive Science Laboratory, Indian Institute of Technology Mandi, Mandi, India; ^2^Defence Terrain Research Laboratory, Defence Research and Development Organization, New Delhi, India

**Keywords:** decision-making, damage-feedback, interactive landslide simulator, reinforcement learning, expectancy-valence model, prospect-valence-learning model

## Abstract

Prior research has used an Interactive Landslide Simulator (ILS) tool to investigate human decision making against landslide risks. It has been found that repeated feedback in the ILS tool about damages due to landslides causes an improvement in human decisions against landslide risks. However, little is known on how theories of learning from feedback (e.g., reinforcement learning) would account for human decisions in the ILS tool. The primary goal of this paper is to account for human decisions in the ILS tool via computational models based upon reinforcement learning and to explore the model mechanisms involved when people make decisions in the ILS tool. Four different reinforcement-learning models were developed and evaluated in their ability to capture human decisions in an experiment involving two conditions in the ILS tool. The parameters of an Expectancy-Valence (EV) model, two Prospect-Valence-Learning models (PVL and PVL-2), a combination EV-PU model, and a random model were calibrated to human decisions in the ILS tool across the two conditions. Later, different models with their calibrated parameters were generalized to data collected in an experiment involving a new condition in ILS. When generalized to this new condition, the PVL-2 model’s parameters of both damage-feedback conditions outperformed all other RL models (including the random model). We highlight the implications of our results for decision making against landslide risks.

## Introduction

Worldwide, landslides cause huge losses in terms of fatalities and injuries and infrastructure damage (Margottini et al., 2011). In fact, landslides and associated debris flows are a major concern for disaster-prevention groups in regions with steep terrains, such as in the Himalayan Mountains (Chaturvedi et al., 2014). Due to the destruction caused by landslides to life and infrastructure, it is essential for people to understand the causes and consequences of landslide disasters as this understanding would likely help people make informed decisions against these disasters. However, prior research suggests that people residing in landslide-prone areas show misconceptions about landslide risks (Oven, 2009; Wanasolo, 2012; Chaturvedi and Dutt, 2015). For example, Chaturvedi and Dutt (2015) evaluated people’s mental models in Mandi, India, a township in the Himalayan Mountains, and one that is frequented by landslides. It was found that residents of Mandi town had a poor understanding of hazard zonation maps of their region. This result is alarming because hazard zonation maps are a common medium for communicating the susceptibility of a region to landslides (Chaturvedi and Dutt, 2015).

Prior research across a number of applied domains shows that interactive simulation tools have been effective in providing the experience of adverse events and also in influencing the understanding and decision making of people living in affected areas (Gonzalez et al., 2005; Knutti et al., 2005; Wagner, 2007; Dutt and Gonzalez, 2011, 2012a; Gonzalez and Dutt, 2011; Sterman et al., 2013; Chaturvedi et al., 2017, 2018). For example, Gonzalez and Dutt (2011) proposed a generic dynamic control task, which was used for investigating people’s decisions in environmental problems. Furthermore, Dutt and Gonzalez (2012a) used this generic control task (called the Dynamic Climate Change Simulator; DCCS) for investigating people’s decisions on climate problems. The DCCS tool provided feedback to people about their decisions and enabled them to reduce their misconceptions about climate change. Similarly, Sterman et al. (2013) have developed a climate change simulation, Climate Rapid Overview and Decision Support (C-ROADS), which can help policymakers and researchers to explore different consequences of carbon emissions policies.

In the area of landslide disasters, Chaturvedi et al. (2017, 2018) have proposed a simulation tool called the Interactive Landslide Simulator (ILS). The ILS tool enables people to make investments against simulated landslides. The probability of occurrence of landslides in ILS is both a function of one’s investment against these disasters as well as other environmental factors (e.g., rain and susceptibility of an area to landslides). Chaturvedi et al. (2017, 2018) used the ILS tool in a laboratory experiment to evaluate how the variations in probability of landslide damages in the ILS influenced people’s understanding of these disasters. It was found that the high probability of damages led to significantly higher investments and understanding compared to the low probability of damages. Chaturvedi et al. (2017, 2018) explained their results based upon the positive and negative experiences gained by people in the ILS tool.

Although the conclusions drawn by Chaturvedi et al. (2017, 2018) are meaningful, these authors did not evaluate their participants’ experiential decisions in ILS by developing models based upon theories of learning from feedback (e.g., reinforcement learning). Such computational models may enable researchers to evaluate the role that certain cognitive mechanisms play in influencing human decisions (Busemeyer and Wang, 2000; Lewandowsky and Farrell, 2011).

In this paper, using the ILS tool, we evaluate human decision making in ILS via cognitive models based upon the theory of reinforcement learning (Sutton and Barto, 1998). Reinforcement learning (referred to as “RL” hereafter) considers people’s decisions to be a function of the positive and negative experiences gained by them in the decision environment (Sutton and Barto, 1998). Thus, RL models seem to be well suited to the ILS tool as people learn from repeated feedback about landslides in this tool. Specifically, we develop computational models relying on RL that attempt to mimic people’s decisions in the ILS tool and, in turn, help us understand the model mechanisms involved in people’s decision making against landslide risks. These model mechanisms may enable researchers to develop preliminary insights into the cognitive processes involved in people’s decision making against landslide risks.

Several RL models have been proposed in the literature to account for human decisions in a few decision environments (Busemeyer and Stout, 2002; Yechiam et al., 2005; Kudryavtsev and Pavlodsky, 2012; Steingroever et al., 2013; Lejarraga and Hertwig, 2016). For example, Steingroever et al. (2013) compared Expectancy Valence (EV), Prospect Valence (PVL), and Expectancy Valence Prospect Utility (EV-PU) models to explain human performance in the Iowa Gambling Task (IGT), where people repeatedly chose between two advantageous and two disadvantageous decks (unknown to people) to maximize payoffs. Kudryavtsev and Pavlodsky (2012) tested three variations of two models, Prospect theory (PT) (Kahneman and Tversky, 1979) and EV (Busemeyer and Stout, 2002), by calibrating model parameters to each participant’s choice. Yechiam et al. (2005) have used the EV model in their study to model brain-damaged subjects, drug-abusers, a special clinical sample (Asperger), and an older-aged sample on the IGT. Furthermore, Lejarraga and Hertwig (2016) have used several RL models to understand the impact of losses on exploratory search in a situation in which exploration was costly. In prior research, mostly RL models have been applied to IGT-like tasks, which are canonical in their make-up. Also, these tasks have involved discrete choice decisions rather than continuous judgments. Overall, the application of RL models to more real-world tasks involving continuous judgments (e.g., monetary investments against landslide disasters) has yet to be fully explored. In this research, we address this gap by evaluating how different RL models account for people’s decision making in the ILS tool, where people make investment decisions against landslides.

The primary objective of this research is to investigate whether mathematical models based upon RL theory and their mechanisms could provide preliminary insights into the cognitive processes that drive participants’ decision making against simulated landslide risks in the ILS tool. For our investigation, we evaluate four RL models namely, the EV model (Busemeyer and Stout, 2002), the PVL model (Ahn et al., 2008), the EV-PU model (Steingroever et al., 2013), and the PVL-2 model (Dai et al., 2015). All of these models work by maximizing the utilities produced by choice on each round. Different from prior research, we develop these models for continuous investment decisions in the ILS tool and evaluate these models’ performance against human data collected with the tool. This paper’s primary objective is to evaluate the ability of EV, EV-PU, PVL, and PVL-2 models and their parameters to account for continuous human decisions in the ILS tool. Furthermore, we interpret the value of calibrated parameters in the best performing RL models to understand human decisions against simulated landsides.

In what follows, first, we provide a brief description of different RL models. Next, we discuss the ILS tool and further detail an experiment in which human participants performed in this tool. Then, we provide a brief explanation of different RL models that we used to evaluate human decision making in the ILS tool. Finally, we fit and generalize different RL models to human decisions in the ILS tool and discuss the role of different RL mechanisms for modeling human decisions in applied domains.

### Reinforcement Learning (RL) Models

The parameters of a cognitive model may provide some insights into the cognitive processes involved when humans make decisions in a task (Daw et al., 2006). For example, Daw et al. (2006) developed different RL models and correlated the mechanisms of these models with findings from functional magnetic resonance imaging for human exploration in a choice task. The prediction error in RL models was correlated significantly with activity in both the ventral and dorsal striatum (Daw et al., 2006). In addition, it has been revealed that the exploration and exploitation processes in RL models are correlated with activity in the dopaminergic, striatal, and medial prefrontal network in the brain (O’Doherty et al., 2004; Bayer and Glimcher, 2005).

The first assumption in RL models is that after each choice in a task, the agent evaluates the rewards and losses associated with the recent choice by means of a utility function. The utility function is then used to calculate the expectancies for the next choice. The option with the highest expectancy value is the most preferred choice. A popular RL model is the Expectancy-Valence Learning model (EV; Busemeyer and Stout, 2002). A modified version of the EV model called the Prospect-Valence Learning (PVL) model (Ahn et al., 2008), accounts separately for losses and gains according to a value function suggested by Prospect Theory (Kahneman and Tversky, 1979; Tversky and Kahneman, 1992). Literature also indicates the use of RL models that combine the EV and PVL models into an Expectancy-Valence with Prospect Utility (EV-PU) model (Ahn et al., 2008). In addition, an alternative version of the PVL model (the PVL-2 model) has been proposed in the literature (Dai et al., 2015). The PVL-2 model is like the PVL model; however, it uses a different utility function. The details of different RL models are discussed next.

### Expectancy-Valence (EV) Model

The EV model was developed by Busemeyer and Stout (2002), and it explains the choice behavior of participants in a task in terms of three psychological processes. The first assumption of the model is that the utility (*V*_*k*_(*t*)) of an option *k* in round *t* is as per the following equation:

(1) $$V_{k}\left(t\right)=\left(1-w\right)*W\left(t\right)+w*L\left(t\right)$$

where *W*(*t*) and *L*(*t*) are the rewards and losses, respectively, on round *t*. The *w* (∈ [0, 1]) is the loss-aversion parameter that considers the weight to losses relative to rewards. A value of *w*greater than 0.5 indicates more weight to losses and loss-averse behavior. A value of *w*less than 0.5 indicates more weight to rewards and reward-seeking behavior. Next, using the utility (*V*_*k*_(*t*)), the participant forms an expectation, *EV*_*k*_, as per the following equation:

(2) $$EV_{k}\left(t+1\right)=\left(1-a\right)*EV_{k}\left(t\right)+a*V_{k}\left(t\right)$$

where *EV*_*k*_(*t*) and *V*_*k*_(*t*) are the expectation and utility of option *k* on round *t*, *EV*_*k*_(0) = 0. The *a* (∈ [0, 1]) is the recency parameter, and it determines the impact of recently experienced utilities or outcomes. A value of *a*greater than 0.5 means that the participants rely on recency, i.e., participants quickly adjust their decisions in response to recent experiences. The EV model assumes that participants use the utility of option *k* on round *t*, i.e., *V*_*k*_(*t*), to update only the expected utility of chosen option *k*, i.e., *EV*_*k*_(*t*+1). The expected utilities of all the other options remain unchanged. Thus, when an option is not chosen in the preceding round, then the option’s EV does not change. Thus, an option’s EV only changes when it is chosen in the last round.

The option with the highest expectancy value is the most preferred one. According to the model, the probability of choosing an option *G_k* is determined by the strength (*EV*_*k*_(*t*))of that option relative to the sum of the strengths of all options as per the following choice rule:

(3) $$Pr\left[G_{k}\left(t\right)\right]=\frac{e^{\left\{\theta \left(t\right)*EV_{k}\left(t\right)\right\}}}{\sum_{k}\left\{e^{\left\{\theta \left(t\right)*EV_{k}\left(t\right)\right\}}\right\}}$$

where *Pr*[*G*_*k*_(*t*)] is defined as the probability that an option *k* will be selected in round *t* by the model. The term θ(*t*), also known as the sensitivity, controls the consistency of choices and depends upon the consistency parameter. The θ(*t*) is defined as:

(4) $$\theta \left(t\right)={\left(\frac{t}{10}\right)}^{c}$$

where *c* (∈ [−5, 5]) is the choice consistency parameter, which determines the extent to which round-by-round choices match the expected utilities of the options. A value of *c* between −5 and 0 indicates strong exploration behavior, whereas a value of *c* between 0 and 5 indicates a strong exploitation behavior. The optimal performance of human-like agents depends on a trade-off between exploration and exploitation. To find the best option, the agent may first explore the choices available in the task. However, if the agent is left with a limited number of rounds, it may be optimal to exploit the option that has produced a maximum profit in the past (Wetzels et al., 2010). Thus, the *c* parameter causes a shift from the exploration behavior to the exploitation behavior over rounds.

### Prospect Value Learning (PVL) Model

The PVL model assumes that humans process the net outcome after a choice, i.e.,

(5) x(t)=W(t)-|L(t)|

where *W*(*t*) and *L*(*t*) are win and loss functions, respectively. The PVL model builds on the EV model, and it uses the prospect-utility function, which is a non-linear utility function from Prospect theory proposed by Tversky and Kahneman (1992). Unlike the EV model, the expectancies of unchosen options are also discounted in the PVL model. The PVL model has four free parameters: shape parameter α, loss aversion parameter (λ), recency parameter (*A*), and consistency parameter (*c*). The α parameter determines the shape of the utility function. The λ parameter determines the attention weight toward losses or rewards. The *A* parameter indicates the effect of recency of outcomes. The *c* parameter determines the amount of exploration vs. exploitation in decision making.

The PVL model has three components. First, the outcome evaluation follows the Prospect utility function, which has diminishing sensitivity to increases in magnitude and different sensitivity to losses vs. gains (i.e., loss aversion). The utility *u*(*t*)on round *t* for each net outcome *x*(*t*) is expressed as:

(6) $$u\left(t\right)=\left\{ \begin{array}{cc} -\lambda {\left|x\left(t\right)\right|}^{\alpha }, & ifx\left(t\right)<0\\ x{\left(t\right)}^{\alpha }, & ifx\left(t\right)\geq 0 \end{array}\right.$$

where 0≤α≤ 1 governs the shape of the utility function, and 0≤λ≤ 5 determines the sensitivity to losses compared to gains. A value of λ≥ 1 indicates that the individual is more sensitive to losses compared to gains. A value of λ <  1 indicates that the individual is more sensitive to gains compared to losses.

Based on the outcome of the chosen option, the expectancies of options are computed using a decay-reinforcement learning rule (Erev and Roth, 1998). In the decay-reinforcement learning rule, the expectancies of all options are discounted with each round, and then the current outcome utility is added to the expectancy of the chosen option,

(7) $$EV_{k}\left(t+1\right)=A*EV_{k}\left(t\right)+\delta_{k}\left(t\right)*u_{k}\left(t\right)$$

The recency parameter 0≤*A*≤1 determines how much the past expectancy is discounted. Here, δ_*k*_(*t*) is a dummy variable, which is 1 if option *k* is chosen and 0 otherwise. The softmax choice rule (Luce, 1959) is then used to compute the probability of choosing an option *j*:

(8) $$Pr\left[G_{k}\left(t\right)\right]=\frac{e^{\left\{\theta \left(t\right)*EV_{k}\left(t\right)\right\}}}{\sum_{k}\left\{e^{\left\{\theta \left(t\right)*EV_{k}\left(t\right)\right\}}\right\}}$$

The θ(*t*) is assumed to be round-independent, and it is set to 3^*c*^−1 (Ahn et al., 2008; Yechiam and Ert, 2007). The *c* parameter (choice sensitivity) varies in the range [0,5].

### Expectancy-Valence Model With Prospect Utility Function (EV-PU)

This model is a combination of the EV and PVL models. It uses the utility function of the PVL model, but all other processes, such as learning rule, choice rule, and sensitivity function, follow the same equations as that of the EV model (Ahn et al., 2008). This construction results in a model with four parameters: (1) The shape parameter (α), (2) the loss-aversion parameter (λ), (3) the recency parameter (*A*), and (4) the response-consistency parameter (*c*).

### Prospect Value Learning-2

The Prospect Value Learning-2 model was proposed by Dai et al. (2015). They compared different combinations of utility functions, learning rules, and choice rules, and the best combination was coined as the PVL-2 model. Dai et al. (2015) proposed a new utility function (PU-2) combining the existing utility function and two learning rules of EV and PVL models, respectively. The alternative prospect utility function challenges the assumption of the prospect utility function of the PVL model. It suggests that when selecting an action that leads to both rewards and losses of the same magnitude, the overall feeling of a participant may not be neutral. For example, the sadness associated with the loss may not be completely offset by the gain. The alternative prospect utility function overcomes this problem by combining features of both the EV and PVL models and is given as follows:

(9) $$u\left(t\right)=win{\left(t\right)}^{\alpha }-\lambda *{\left|loss\left(t\right)\right|}^{\alpha }$$

where *win*(*t*) and *loss*(*t*) are the amounts of rewards and losses, and α and λparameters are the same as those in the PVL model. The learning rule and the choice rule remain unchanged from the PVL model in the PVL-2 model. In summary, the PVL-2 model also has four parameters: shape parameter α, loss-aversion parameter λ, recency parameter *A*, and consistency parameter *c*. Their interpretations and ranges remain unchanged from the PVL model.

### Random Model

To compare the performance of RL models, a baseline random model having no learning was created. In this model, the expectancies associated with all choices were computed independently and randomly in a range [0, 1]. The decision choice corresponding to the highest expectancy was executed in a round. This model assumes no parameters.

Dai et al. (2015) compared 18 models on a population of 26 opiate users and found two key results: first, the learning rule of the EV model was always inferior to that of the PVL model; and, second, the prospect utility function of PVL-2 performed better than the utility functions of both EV and PVL models. While these RL models have been helpful in understanding neuropsychological aspects of clinical populations in IGT and SGT, these tasks are simple, involve discrete choices, and are mostly disconnected from real-world situations. Overall, there is a need for evaluating RL models in real-world tasks, and this evaluation will help us understand the potential of different RL models in applied real-world domains.

The primary objective of this paper is to evaluate the ability of EV, EV-PU, PVL, PVL-2, and random models, and their parameters to account for human decisions in a complex interactive landslide simulator tool. Furthermore, we generalize the calibrated RL models to understand their ability to account for human decisions against simulated landsides in a novel dataset. Also, the calibrated RL models are compared to a baseline random model with no learning.

### The Interactive Landslide Simulator (ILS) Tool

**FIGURE 1:**
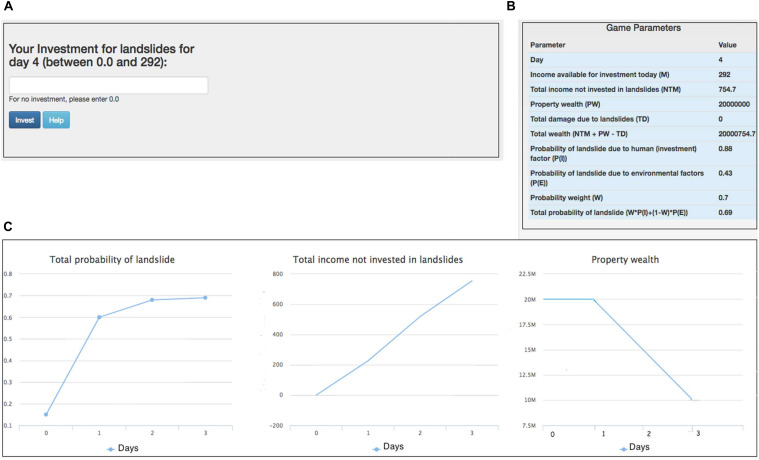
The Investment Screen in ILS tool. **(A)** The text box where participants made investments against landslides. **(B)** The tool’s different parameters and their values. **(C)** Line graphs showing the total probability of landslide, the total income not invested in landslides, and the property wealth over days. Horizontal axes in these graphs represents number of days. The goal was to maximize the Total Wealth across a number of rounds of performance in the ILS tool. This figure is adapted from Chaturvedi et al. (2017).

Recent research has proposed the use of an ILS tool to test how people make decisions against landslide risks when people are provided with different amounts of damage feedback (Chaturvedi et al., 2017, 2018). In the ILS tool, people are asked to make monetary investment decisions (see Figure 1), where people’s monetary contributions would be used for mitigating landslides (e.g., by building a retaining wall, planning road construction, or provisioning proper drainage).

The goal of the ILS tool is to maximize one’s total wealth, where this wealth is influenced by one’s income, property wealth, and losses experienced due to landslides. The ILS tool considers both environmental factors (spatial geology and rainfall) and human factors (people’s investments against landslides) for calculating the probability of landslide occurrence. Once people make monetary decisions in the ILS tool, the tool provides feedback on whether a landslide occurred and whether there was a reduction in income (due to injury or fatality) or a reduction in property wealth (due to property damage). As shown in Figure 1, people are also shown different variables on the graphical user interface as well as plots of the total probability of landslides, income not invested in landslides, and property wealth. As described by Chaturvedi et al. (2017, 2018), the total probability of landslides in the ILS tool is a function of landslide probabilities due to human factors and physical factors. This total probability of landslides can be represented as the following:

(10) P(T)=(We*P(I)+(1-We)*P(E))

where *We* is a free weight parameter in [0, 1]. The total probability of landslides involves the calculation of two probabilities, probability of landslide due to human investments (*P(I)*) and the probability of landslide due to physical factors (*P(E)*).

### Probability of Landslide Due to Human Investments

As suggested by Chaturvedi et al. (2017, 2018), the probability *P*(*I*) is calculated using the probability model suggested by Hasson et al. (2010). In this model, *P*(*I*) is directly proportional to the amount of money invested by participants for landslide mitigation. The probability of landslide due to human investments is:

(11) $$P\left(I\right)=1-\frac{M*\sum_{i=1}^{n}x_{i}}{n*B}$$

where *B* is the income available for mitigating landslides in a round, *n* is the number of rounds (days, months, or years as set in the simulation), *x*_*i*_ is the investments made by a person for mitigating landslides in the round *i* (x_*i*_ ≤ B), and *M* (0 ≤ M ≤ 1) is the return to mitigation parameter, which is a free parameter that accounts for the lower bound probability of *P(I)*, i.e., *P (I) = 1 – M* when a person puts his entire budget B into landslide mitigation ($\sum_{i=1}^{n}x_{i}$ = n*B). People’s monetary investments (*x*_*i*_) against landslides are for promoting mitigation measures like building retaining walls or planting long root plants. The ILS model currently assumes that human investments may still not suffice to reduce the *P*(*I*) to 0 (because 1−*M* > 0). That is because human interventions against landslides may not be fool proof. In contrast, if a participant does not invest in landslide mitigation, then the *P*(*I*) would increase rapidly.

### Probability of Landslide due to Physical Factors

Some of the physical factors impacting landslides include rainfall, soil types, and slope profiles (Chaturvedi et al., 2017, 2018). These factors can be categorized into two parts:

1. Probability of landslide due to rainfall [*P(R)*]
2. Probability of landslide due to soil types and slope profiles [spatial probability, *P(S)*]

The *P(R)* is determined based upon the daily rainfall profile of the study area, and *P(S)* is determined by the susceptibility of the study area to experience landslides due to soil types and slope profiles (Chaturvedi et al., 2017, 2018).

In the ILS tool, if a uniformly distributed random number [∼*U(0, 1)*] is less than or equal to *P(T)* (Eq. 10) in a certain round, then a landslide occurs in that round. When a landslide occurs, it may be benign or damaging. A landslide is damaging if it causes injury, fatality, or property damage, where each of these causes is independent of each other, and they can get triggered independently by pre-defined point probabilities. If none of these damages occurs when a landslide occurs in a round, then the landslide is termed as benign.

### Calibration Dataset and ILS Parameters by Chaturvedi et al. (2017, 2018)

Chaturvedi et al. (2017, 2018) defined two between-subjects damage-feedback conditions in the ILS tool: low damage (*N* = 20) and high damage (*N* = 23). Participants were randomly assigned to these two conditions, and all participants in the study were from Science, Technology, Engineering, and Mathematics (STEM) backgrounds. Ages ranged between 21 and 28 years (Mean = 22 years; Standard Deviation = 2.19 years). All participants received a base payment of INR 50 (∼USD 1) after completing their study. In addition, there was a performance incentive-based upon a lucky draw. Based upon total wealth remaining at the end of the study, top-10 performing participants were put in a lucky draw, and one of the participants was randomly selected and awarded a cash prize of INR 500. Participants were told about this performance incentive before they started their experiment. Each condition was 30 rounds long, where, in each round, participants made monetary investment decisions to mitigate landslides and observe the consequences of their decisions (Chaturvedi et al., 2017, 2018).

Table 1 shows the values of different parameters in the two conditions. As shown in Table 1, in the high damage-feedback condition, the probabilities of property damage, fatality, and injury on any round were set at 30, 9, and 90%, respectively. In the low damage-feedback condition, the probabilities of property damage, fatality, and injury on any round were set at 3, 1, and 10%, respectively (i.e., about 1/10th of its values in the high condition). The proportion of damage (in terms of daily income and property wealth) that occurred in the event of fatality, injury, or property damage was kept constant across 30 rounds. The property wealth decreased to half of its value every time property damage occurred in the event of a landslide. The daily income was reduced by 10% of its latest value due to a landslide-induced injury and 20% of its latest value due to a landslide-induced fatality. The initial property wealth was fixed to 20 million EC, which was the expected property wealth in the study area. To avoid the effects of currency units on people’s decisions, we converted Indian National Rupees (INR) to a fictitious currency called “Electronic Currency (EC),” where 1 EC = 1 INR. The initial per-round income was kept at 292 EC (taking into account the GDP and per-capita income of the Himachal state where the study area was located). The weight (*We*) parameter in Eq. 10 was fixed at 0.7 across all conditions.

**TABLE 1:** Parameters across different conditions of Chaturvedi et al. (2017, 2018).

Parameters	Low condition (calibration condition)	High condition (calibration condition)
Initial income	292 EC^*a*^	292 EC
Initial property wealth	20 million EC	20 million EC
*We* parameter	0.7	0.7
Probability of property damage	03%	30%
Probability of injury damage	10%	90%
Probability of fatal damage	01%	9%
A loss to property wealth due to property damage	50% of available property wealth	50% of available property wealth
A loss to income due to injury	10% of available income	10% of available income
A loss to income due to fatality	20% of available income	20% of available income

**^*a*^Chaturvedi et al. (2017, 2018) used a fictitious currency called “EC.”**

Data collected in the two conditions were analyzed in terms of the average investment ratio. The investment ratio was defined as the ratio of total investments made by participants up to a round divided by the total investments that could have been made up to the round. The investment ratio was averaged over all participants for a round as well as averaged over all participants and rounds. Given the effectiveness of feedback in simulation tools (Gonzalez et al., 2005; Sterman, 2011; Dutt and Gonzalez, 2012a; Chaturvedi et al., 2017, 2018) expected participant investments to be greater in the high condition compared to the low condition. According to Chaturvedi et al. (2018), the average investment ratio was significantly higher in the high condition (0.67) compared to that in the low condition (0.38) [*F*(1, 41) = 17.16,*p* < 0.001,η^2^ = 0.29]. Furthermore, the investment ratio in the high condition increased rapidly compared to that in the low condition across rounds [*F*(6.25, 256.4) = 7.53,*p* < 0.001,η^2^ =  0.16]. Overall, the high damage-feedback in ILS helped participants to increase their investments for landslide mitigation (Chaturvedi et al., 2018).

The data collected in the two conditions by Chaturvedi et al. (2018) was used to calibrate different RL models in this paper. For more details on experimental design, participant, procedure, and behavioral results, please refer to Chaturvedi et al. (2018).

### Generalization Dataset and ILS Parameters

For this paper, a new study was performed involving the ILS tool to test the generalizability of the calibrated RL models. In the new study, participants could voluntarily participate in the ILS tool in a “medium” damage-feedback condition, where the ILS parameters were defined to be in between those in the low and high damage-feedback conditions. Table 2 shows the ILS parameters used in the medium condition in the new experiment. As shown in Table 2, in the medium condition, the probabilities of property damage, fatality, and injury in any round were set at 16.5, 5, and 50%, respectively (i.e., in between their values in the high and low feedback-damage conditions). The other parameter values were the same as those in the study by Chaturvedi et al. (2017, 2018).

**TABLE 2:** Parameters across the medium condition in the interactive landslide simulator (ILS) tool.

Parameters	Medium condition (generalization condition)
Initial income	292 EC
Initial property wealth	20 million EC
Probability weight (*We*)	0.7
Probability of property damage	16.5%
Probability of injury damage	50%
Probability of fatal damage	5%
A loss to property wealth due to property loss	50% of available property wealth
A loss to income due to injury loss	10% of available daily income
A loss to income due to a fatal loss	20% of available daily income

The data collected in the medium condition in Table 2 was used to generalize different models in this paper.

### Participants and Procedure

The study was approved by the Ethics Committee at the Indian Institute of Technology (IIT) Mandi. Thirty participants were recruited at IIT Mandi via an online advertisement. Informed consent was obtained from each participant before the beginning of the study, and participation was completely voluntary. Participants were from STEM backgrounds, and their ages ranged between 23 and 45 years (Mean = 28.67 years; Standard Deviation = 5.95 years). There were 56.67% males, and the rest were females. All participants received a base payment of INR 50 (∼USD 1). In addition, there was a performance incentive-based upon a lucky draw. Top-10 performing participants based upon total wealth remaining at the end of the study were put in a lucky draw, and one of the participants was randomly selected and awarded a cash prize of INR 500. Participants were told about this performance incentive before they started their experiment. The medium condition was 30 rounds long, where, in each round, participants made monetary investment decisions to mitigate landslides and observe the consequences of their decisions. At the start of the experiment, participants read instructions, and once ready, started their study. Upon completing the study, participants were thanked and paid for their participation.

### Distribution of Investment Ratios and Parameter Calibration

In ILS, human and model participants were evaluated based upon their investment ratios. To make the discrete RL models generate continuous investment ratios, we made each model to choose between *10* decision options, where each option was mapped to a bin of 10% investment-ratio width. Thus, for each model, *10* decision options were defined at each round (*k* = 1, 2, 3,, 10) to choose between and each decision option mapped to a 10% investment ratio bin. So, if a model chose the first decision option (*k* = 1), then the model suggested an investment ratio in the range [0, 10%]. Similarly, if the model chose the second decision option (*k* = 2), then the model suggested an investment ratio in the range (10, 20%) and so on. Once the bin was chosen based upon the decision option, a random number in the bin range was chosen as the model’s investment ratio decision. For example, if a model participant chose the first decision option at a round *t*, then a random number in the range (0, 10%) was selected as the investment ratio decision in that round. Similarly, if a model participant chose the sixth decision option in a round *t*, then a random number in the range (50, 60%) was selected as the investment ratio decision in that round. While considering the bin of 10% investment-ratio width, we considered a number of other ways to choose between different decision options. For example, one way may be to consider the investment ratio as the maximizing probability value in a round for a decision option. However, if one equated this maximizing probability value as the investment ratio, then doing so may yield unrealistically high values of the investment ratios across rounds. These investment ratios would be unlike the investment ratios exhibited by participants in the ILS tool. Overall, the 10% binning method suggested above would allow investment ratios to vary over a large range, and thus this method was adopted to calibrate models to human data.

### Reward Functions in RL Models

In each model, the *W*(*t*) (win) function and *L*(*t*) (loss) function were defined in the following manner:

(12) $$W\left(t\right)=income_{t}-invest_{t}$$

(13) $$L\left(t\right)=PD_{t}+\left(income_{t-1}-income_{t}\right)$$

where *income*_*t*_ was the income obtained by the player in each round (in the human experiment, each round earned an income amount). The *invest*_*t*_ was the investment made by the model in the round *t*. The *PD*_*t*_ is the property damage due to a landslide and (*income*_*t*−1_−*income*_*t*_)is the decrease in income from the last round to the current round due to an injury or fatality in a landslide. The *W*(*t*) and *L*(*t*) defined in Eqs 12 and 13 were used in different RL models as the reward and loss functions, respectively.

### Model Execution and Evaluation

All RL models were created in Excel^®^, and these models possessed different free parameters as discussed above. The model parameters were calibrated using the same number of simulated participants as the number of human participants that participated in the experiment. The initial expectations [*EV*_*k*_(0)]across all choices (investment bins) were assumed to be equal to 50 units in each model. As the value of expectations was equal (=50), the first decision choice for a bin was made randomly in each RL model. As the same value of 50 units was assumed across all models for the initial expectations, these initial expectations were not considered as free parameters in model calibrations. Two separate calibrations were performed, one to each of the two damage-feedback conditions, where the models were evaluated on the sum of squared deviation (SSD1) and *R*^2^ (both computed over rounds). The SSD1 accounted for the error between the model’s investment ratio and human’s investment ratio over 30-rounds, and it was calculated as:

(14) $$SSD1=\frac{1}{30}\sum_{t=1}^{30}{\left(M_{t}-H_{t}\right)}^{2}$$

where *M_t* and *H_t* refer to the average investment ratio from the model and human data in round *t*, respectively (the average was taken across all models and human participants for each round). A smaller value of *SSD1* was desirable as it meant a smaller error between model and human investment ratios.

The square of the Pearson’s correlation coefficient(*R*^2^) indicated the model’s ability to account for the trend in human data. The higher the value of *R*^2^ (closer to 1.0), the better was the model’s ability to account for the trend in human data compared to an average model. The *R*^2^ was defined over 30-rounds as:

(15) $$R^{2}=\frac{30\sum_{t=1}^{30}\left(M_{t}*H_{t}\right)-\sum_{t=1}^{30}M_{t}*\sum_{t=1}^{30}H_{t}}{\sqrt{\begin{array}{c} \left[30*\sum_{t=1}^{30}M_{t}^{2}-{\left(\sum_{t=1}^{30}M_{t}\right)}^{2}\right]\\ {}*\left[30*\sum_{t=1}^{30}H_{t}^{2}-{\left(\sum_{t=1}^{30}H_{t}\right)}^{2}\right] \end{array}}}$$

Since *SSD1* was to be minimized and *R*^2^ was to be maximized, the sum of *SSD1* and 1−*R*^2^ was minimized as the objective function for parameter calibration across different models. The genetic algorithm (Konak et al., 2006) was used for minimizing the sum of *SSD1* and 1−*R*^2^. The multi-objective genetic algorithm varied the values of parameters for the simulated model in the ILS task to minimize the objective function. The parameters were adjusted over their defined range to ensure that the optimization was able to capture the optimal parameter values in their respective ranges with high confidence. The genetic algorithm had a population size of 20, a crossover rate of 80%, and a mutation rate of 1%. The algorithm stopped when any of the following constraints were met: stall generations = 50, function tolerance = 1 × 10^–8^, and the average relative change in the fitness function value over 50 stall generations was less than function tolerance (1 × 10^–8^). These assumptions are similar to other studies in literature where models have been fitted to human data using the genetic algorithm (Gonzalez and Dutt, 2011; Sharma and Dutt, 2017).

As different RL models possessed different parameters, the ordinary least squares (OLS) formulation of the Akaike Information Criterion (AIC) was used to evaluate the performance of different RL models (Dutt and Gonzalez, 2011; Derryberry, 2014; Banks and Joyner, 2017). The AIC incorporated both a model’s ability to predict human data (error) and the model’s complexity (number of free parameters). Different AICs were defined by the following formulae:

(16) AIC1=30*ln(SSD1)+2*p

(17) $$SSD2={\left(X_{model}-X_{human}\right)}^{2}$$

(18) AIC2=ln(SSD2)+2*p

where *AIC1* was computed using the investment ratio that was averaged over human or model participants in each round. *AIC2* was computed using the investment ratio that was averaged over both human or model participants and rounds. The *p* is the number of free parameters in a model. The *X*_*model*_ and *X*_*human*_ refer to the average investment ratio of the model and human participants, respectively, where the average was taken over all participants and rounds. The smaller or more negative the value of AICs, the better the respective model.

### Expectations From Models

As discussed above, prior research has relied upon RL theories for understanding and accounting for the decisions made by people in dynamic tasks (Arora and Dutt, 2013; Dutt and Kaur, 2013; Dutt et al., 2013). According to these theories, human players would tend to rely upon recency of outcomes, and players would choose those actions that maximize their utility (Steingroever et al., 2013; Gonzalez et al., 2015). For example, in ILS, participants may experience deaths, injuries, and property damages due to landslides across both the high and low conditions. When the damage is low in ILS, participants, relying upon the recency of experienced outcomes, may choose to conservatively invest their income in landslide mitigation. That is because by investing smaller incomes, they may maximize their total earnings. However, when the damage is high in ILS, participants, again relying upon the recency of experienced outcomes, may likely increase their investments to fight landslides. Overall, participants are likely to be influenced by the recency of experienced outcomes.

We expected different RL models to perform better in accounting for human participants’ decisions in ILS than the baseline random model with no learning. Also, we expected the PVL models to perform better compared to other RL models. One likely reason for this expectation is due to the nature of the prospect utility function in the PVL model, where this function accounts for both losses and rewards non-linearly. Another likely reason for this expectation is because the PVL models incorporate two parameters (α, λ) that collectively describe sensitivity to rewards and losses; whereas, the EV model computes outcome utilities based upon a single parameter (*w*). Next, we test these expectations by calibrating and generalizing all RL models to human data in the ILS tool.

### Model Calibration Results

Table 3 shows the performance in terms of SSD1, *R*^2^, AIC1, and AIC2 values across different RL and random models in the calibration conditions in the ILS tool. As shown in Table 3, based on the *R*^2^, the PVL model performed the best in the low and high conditions. Based upon R^2^, the PVL model was followed by the PVL-2 model (low damage-feedback) and EV-PU model (high damage-feedback). The PVL model also possessed the third most negative and the most negative AIC1 in low and high conditions, respectively. Table 3 also shows that when performance was compared between models and human data by averaging over participants and rounds, the AIC2 values were the best (lowest) for the following models: random and EV-PU models in low condition and random and EV models in the high condition.

**TABLE 3:** Performance of expectancy-valence (EV), expectancy valence-prospect utility (EV-PU), prospect-valence learning (PVL), PVL-2, and random models in the high and low conditions in the ILS tool.

Model	Low				High			
	SSD1	*R*^2^	AIC1	AIC2	SSD1	*R*^2^	AIC1	AIC2
EV	0.02	0.003	–113.52	1.77	0.05	0.11	–79.97	2.45
EV-PU	0.05	0.005	–80.73	0.87	0.06	0.68	–73.87	5.03
PVL	0.02	0.17	–110.64	2.03	0.02	0.81	–109.03	2.85
PVL-2	0.10	0.15	–60.18	5.48	0.05	0.58	–78.46	4.67
Random	0.01	0.021	–126.50	–4.33	0.06	0.02	–84.29	–3.53

**FIGURE 2:**
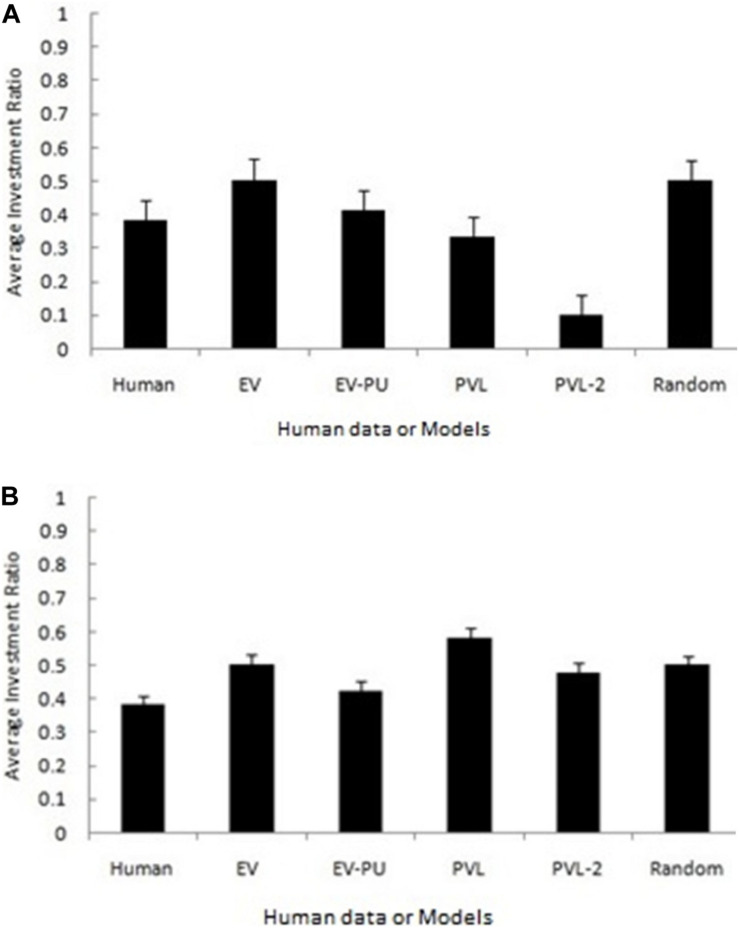
The average investment ratio (averaged over participants and rounds) in human data, reinforcement learning (RL) models, and the baseline random model in low condition **(A)** and high condition **(B)**. The error bars show the 95% confidence interval around the point estimate.

Figures 2A,B show the predicted average investment ratios (averaged over participants and rounds) from different RL models, random model, and human data in the low and high conditions, respectively. Overall, a high-level aggregation across participants and rounds seemed to make even the poor performing models (e.g., random and EV models) look good.

**FIGURE 3:**
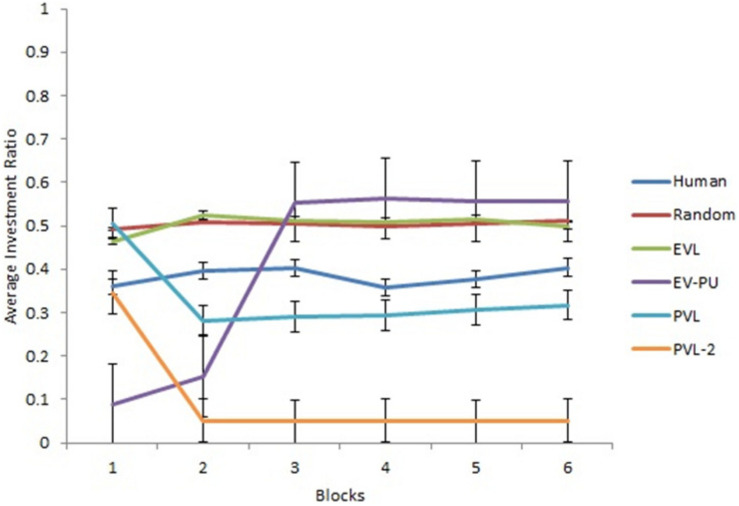
Investment ratio from different RL models, random model, and human data across 6 blocks of 5 rounds in the low condition. Error bars show a 95% confidence interval around the point estimate.

**FIGURE 4:**
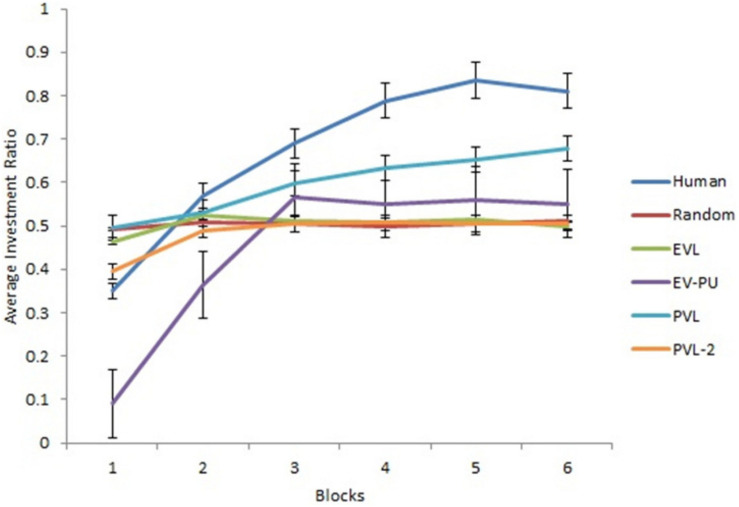
Investment ratio from different RL models, random model, and human data across six blocks of five rounds in the high condition. Error bars show a 95% confidence interval around the point estimate.

Next, we analyzed the predictions from different RL models’ over 30 rounds in the two calibration conditions. To reduce the degree of freedom across rounds, we converted the rounds into blocks such that there were six blocks of five rounds each (i.e., the value of five rounds was averaged in each block). Figures 3, 4 show the comparison of human data with data from different RL models across the six blocks in low and high conditions, respectively. Based on R^2^ and AIC1 values, the PVL and PVL-2 models (low damage-feedback) and the PVL and EV-PU (high damage-feedback) were the best performing models over blocks.

### Model Generalization Results

The RL models with their calibrated parameters in the low and high conditions were generalized to human data collected in the medium condition. Table 4 shows the performance of RL models with calibrated parameters of the high condition and low condition when generalized to the medium condition. Based upon the *R*^2^, AIC1, and AIC2 values, the PVL-2 model performed the best in predicting the human investment ratios in the medium condition across both its parameters from the low and high conditions. Also, all RL models performed better compared to the baseline random model in the generalization. Only the AIC1 value of the EV-PU model was worse compared to the random model when the EV-PU model’s parameters were calibrated in low damage-feedback condition. Overall, these results are as per our expectations.

**TABLE 4:** A comparison of EV, EV-PU, PVL, PVL-2, and random models’ performance in the medium condition with calibrated parameters of low and high conditions in ILS.

Model	Parameters calibrated in low				Parameters calibrated in high			
	SSD1	*R*^2^	AIC1	AIC2	SSD1	*R*^2^	AIC1	AIC2
EV	0.006	0.58	–153.50	–5.81	0.007	0.74	–146.56	–5.12
EV-PU	0.015	0.31	–125.49	–4.54	0.005	0.75	–154.82	–5.62
PVL	0.002	0.72	–179.25	–7.13	0.003	0.70	–169.43	–6.45
PVL-2	0.002	0.73	–184.89	–7.67	0.002	0.70	–181.87	–7.83
Random	0.010	0.001	–136.67	–5.50	0.010	0.001	–136.67	–5.51

*The number of free parameters was taken to be 0 in the Akaide Information Criterion (AIC) values for all models as the free parameters were fixed to their calibrated values in the generalization.*

**FIGURE 5:**
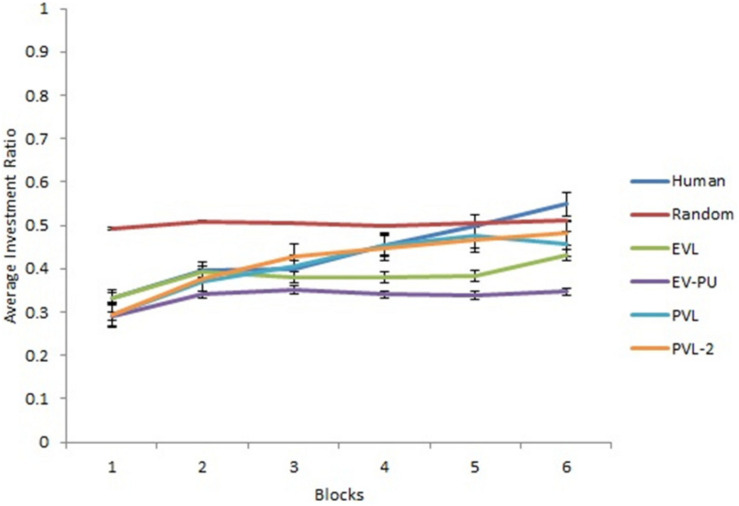
Investment ratio from different RL models calibrated in the low condition, random model, and human data across six blocks of five rounds in the medium condition. Error bars show a 95% confidence interval around the point estimate.

**FIGURE 6:**
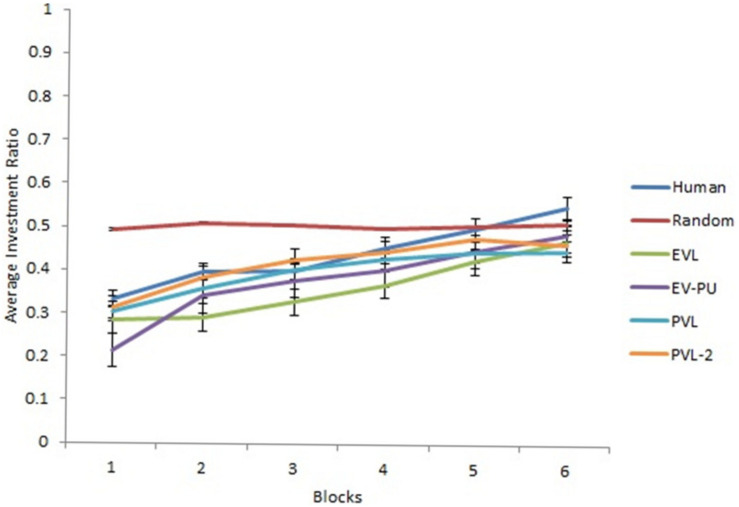
Investment ratio from different RL models calibrated in high condition, random model, and human data across six blocks of five rounds in the medium condition. Error bars show a 95% confidence interval around the point estimate.

Based on *R*^2^ and AIC1 values in Table 4, the PVL-2 performed the best among all models during generalization. Figures 5, 6 show the predicted average investment ratios from different RL models (calibrated in low and high conditions, respectively), random model, and human data in the medium condition across blocks.

### Calibrated Parameters

Table 5 shows the calibrated values of parameters across the EV, EV-PU, PVL, PVL-2, and random models in the low and high conditions of ILS, respectively. The best set of parameters (corresponding to the PVL-2 model) have been italicized.

**TABLE 5:** Calibrated model parameters of EV, EV-PU, PVL, PVL-2, and random models in the high and low damage conditions in ILS.

Model	Low damage	High damage
EV	a = 0.771, w = 0.079, c = 0.282	a = 0.115, w = 0.728, c = 0.819
EV-PU	α = 0.931, λ = 1.000, c = 1.000, A = 1.0	α = 0.880, λ = 0.129, c = −0.836, A = 0.954
PVL	α = 0.557, λ = 2.204, c = 0.311, A = 0.748	α = 0.851, λ = 4.797, c = 0.789, A = 0.099
*PVL-2*	*α = 0.557, λ = 2.204, c = 0.311, A = 0.748*	*α = 0.851, λ = 4.797, c = 0.789, A = 0.099*
Random	No parameters	No parameters

*The best-fitting PVL-2 model parameters based upon the generalization have been italicized.*

As shown in Table 5, in the PVL-2 model, the α parameter was closer to one in both damage-feedback conditions (more in the high damage compared to low damage), and this parameter indicated that the shape of the participant’s utility function was curved with diminishing marginal utility and the utility increased proportionally to the net outcome *x*(*t*) in the ILS tool. Second, the value of λ parameter (i.e., sensitivity to losses compared to rewards) was higher in the high damage-feedback condition compared to the low damage-feedback condition, and this meant that people gave more weight to losses in the high damage-feedback condition compared to low damage-feedback condition (in fact, losses were higher in the high damage-feedback condition). Overall, participants acted loss-averse as the median value of the λ parameter was 3.50 (average of 2.204 and 4.797), i.e., more than 1. Third, the PVL model showed a strong influence of recency among participants’ decisions in the high damage-feedback condition (the *A* parameter closer to 0) compared to those in the low damage-feedback condition (the *A* parameter closer to 1). This result is as per our expectation, where the high damage-feedback condition was expected to create more recency effects compared to the low damage-feedback condition. Finally, the consistency parameter (*c*) value was higher in the high damage-feedback condition compared to that in the low damage-feedback condition, showing more exploitation in the high damage-feedback condition compared to the low damage-feedback condition. However, the *c* parameter’s value was closer to 0 across both conditions, and thus mostly participants’ investment decisions were explorative in the ILS tool.

## Discussion

Due to landslide risks in hilly areas the world over, it is important to evaluate people’s mitigation decisions against these disasters. In the absence of participant interviews, computational models that rely upon theories of cognition (e.g., reinforcement learning or RL) may help predict people’s mitigation decisions in situations involving landslide risks. The primary goal of this research was to meet this objective and compare the ability of a number of RL models to account for people’s mitigation decisions against landslides in an interactive landslide simulator (ILS) task involving continuous investment decisions. The parameters of the best fitting PVL-2 model would help to infer the cognitive states of participants in the ILS tool. An experiment involving the ILS tool was reported that investigated participants’ decision making in two different damage-feedback conditions: the high and the low damage-feedback (Chaturvedi et al., 2017, 2018). The two conditions produced different learning curves: The high damage-feedback condition showed much greater learning and a different direction of learning compared to the low damage-feedback condition. These empirical results provided the data sets for calibrating RL models. Furthermore, a new experiment was conducted with ILS in a medium damage-feedback condition for generalization of calibrated RL models. In this paper, we built on ILS’s experimental work and developed computational RL models that could account for people’s decision making in conditions involving different damage feedback in ILS.

First, our results showed the superiority of the PVL-2 model over other RL models and a random model about these models’ ability to explain the actual choice behavior in the ILS tool accurately. In fact, the PVL-2 model also performed well in its generalization to the medium damage-feedback condition collected in the ILS tool. Overall, our results agree with those of Ahn et al. (2008) and Dai et al. (2015), who showed that the prospect utility function had better accuracy and generalizability than the expectancy utility function when accounting for participants’ choices in simple decision making tasks (e.g., IGT).

Second, our results suggested that the loss-aversion (λ*)* parameter is much higher in the high damage-feedback condition compared to the low damage-feedback condition. This meant that people tended to give more weight to losses compared to rewards, especially when losses occurred a greater number of times. The PVL-2 model’s parameters also showed a greater reliance on recency of outcomes for participants in the high damage-feedback condition compared to those in the low damage-feedback condition. A likely reason for this observation could be that the participants acted risk-averse and invested more when recent damages in ILS were high compared to low. In the high damage-feedback condition, losses occurred frequently, and that is perhaps why people start paying attention to these losses. The consistency parameter’svalue was also larger in the high damage-feedback condition compared to that in the low damage-feedback condition. One likely reason for this parameter value could be that people in the high damage-feedback condition were more exploitative in their choices compared to those in the low damage-feedback condition. In contrast, in the low damage-feedback condition, the value of the consistency parameter was low, and participants showed more explorative behavior. Group differences on this parameter may reflect a tendency to seek out and engage in risky experiences in the low damage-feedback condition compared to those in the high damage-feedback condition. Furthermore, we found that participants’ utilities were influenced by net outcomes in the high damage-feedback condition compared to the low damage-feedback condition. One likely reason for this finding could be that participants in the low damage-feedback condition suffered fewer losses due to landslides compared to the participants in the high damage-feedback condition. It could be that the perception of smaller losses among participants in the low damage-feedback condition made the PVL-2 model disregard the net outcome in the experiment.

Although we trained and tested RL-based mathematical models on datasets collected in lab-based experiments, the proposed RL models could help create computer agents that perform like human participants in real-world landslide scenarios. Here, these model agents could help predict the decision making of human participants in some of the landslide scenarios.

There are a number of things to explore as a part of future research. First, we assumed a constant explore/exploit parameter that did not change over rounds. In the future, it may be worthwhile to vary this parameter to obtain more realistic results. Furthermore, there are likely to be individual differences among participants in the ILS tool. Thus, in the future, it would be interesting to model individual human performance compared to aggregate human performance. The analyses at the individual level could result in different investment ratio trajectories than the almost flat line obtained currently in the low condition. As the RL models did not perform well in some of the landslide scenarios in this paper, it may also be worthwhile to evaluate how models built using other advanced cognitive theories (e.g., Instance-based Learning; Dutt and Gonzalez, 2012b) would perform in these scenarios. Also, it may be worthwhile to calibrate RL models to data in the ILS tool involving the presence of social norms and differing amounts, availability, format, and speed of feedback. Some of these ideas form the immediate next steps that we plan to undertake in our research program in the understanding of human decision making against landslide risks.

## Conclusion

The ILS tool incorporates cognitive and motivational processes (responses to rewards and losses) associated with the anticipation of outcomes following investment decisions over time. To perform well in the ILS tool, participants need to learn on the basis of rewards and losses experienced in the task. Consistent with prior literature (O’Doherty et al., 2004; Bayer and Glimcher, 2005; Daw et al., 2006), computational cognitive models and their mechanisms may allow us to get preliminary insights about the cognitive processes contributing to ILS performance. Our analyses revealed that the PVL-2 model accounted for participants’ behavior in the ILS tool more accurately compared to other RL models. This result may be due to the PVL-2 model’s use of a prospect utility function, which can account for the subjective evaluation of rewards/losses, recency effects, and decreasing sensitivity to larger vs. smaller rewards (Ahn et al., 2008). Overall, based upon cognitive modeling, we found that human decisions in the ILS tool to be driven by outcomes, loss-aversion, reliance upon recent outcomes, and exploratory behavior. Future investigations may focus on finding similarities and differences with reported results across diverse populations. Collectively, this knowledge may contribute to the development of preventive approaches for landslide risk reduction that are sensitive to individual differences due to specific processes underlying decision making.

## Data Availability Statement

The datasets generated for this study are available on request to the corresponding author.

## Ethics Statement

The studies involving human participants were reviewed and approved by the Ethics Committee at the Indian Institute of Technology Mandi. The patients/participants provided their written informed consent to participate in this study.

## Author Contributions

PC developed the ILS tool and RL models under the guidance of VD. PC and VD collected the data in the study and calibrated the model parameters, analyzed the data, and prepared the manuscript. Both authors contributed to the article and approved the submitted version.

## Conflict of Interest

The authors declare that the research was conducted in the absence of any commercial or financial relationships that could be construed as a potential conflict of interest.
